# Eco-social and behavioural determinants of diarrhoea in under-five children of Nepal: a framework analysis of the existing literature

**DOI:** 10.1186/s41182-016-0006-9

**Published:** 2016-04-03

**Authors:** Shyam Sundar Budhathoki, Meika Bhattachan, Ajay Kumar Yadav, Pawan Upadhyaya, Paras K. Pokharel

**Affiliations:** School of Public Health and Community Medicine, B.P. Koirala Institute of Health Sciences, Dharan, Nepal; Department of General Practice and Emergency Medicine, B.P. Koirala Institute of Health Sciences, Dharan, Nepal

**Keywords:** Diarrhoea, Under five, Nepal, Dahlgren and Whitehead, Eco-social

## Abstract

**Background:**

While diarrhoea is the second major killer among the under-five children in the world with an estimation of 760,000 deaths annually, it stands as a major killer in Nepal with an annual incidence of 500 per 1000 under-five children with diarrhoea. Diarrhoea is responsible for a wide range of morbidity and mortality among children in Nepal. The objective of this review work is to identify the eco-social and behavioural determinants of diarrhoea among the under-five children of Nepal.

**Methods:**

A literature review was conducted using the Dahlgren and Whitehead model (1991) between June and October 2015. PubMed, Nepal Journals online and Google Scholar were used to search for literature published between 1989 and July 2015 using defined keywords.

**Results:**

Children of age group 6–23 months are at higher risk, as supplementary diets are introduced to the children from the age of 6 months. Male children have better access to healthcare services. Malnourished children also have a higher chance of developing persistent diarrhoea. Provision of safe water and sanitation has direct link with the prevention and control of diarrhoea. Male gender with high income positively influences the treatment-seeking behaviour. Mother’s education and hand-washing practice have direct influence in child health. Hand-washing practices with soap which are protective are influenced by the cultural beliefs. Involvement of community health volunteers increases the access to the health system, thereby reducing the diarrhoeal burden in the community.

**Conclusion:**

Age, gender, hand-washing behaviour, nutritional status of children, education of mothers, water and sanitation, healthcare services, cultural and societal values and income of the household were identified determinants for diarrhoea in under-five children of Nepal.

## Background

Diarrhoea is defined as the passage of the loose or watery stool at least three or more times in a day. However, consistency of the stool is more important rather than the frequency [1]. Globally, there are around 1.7 billion cases of diarrhoeal disease every year and is the second major killer among the under-five children in the world with an estimation of 760,000 deaths annually [2] accounting for one tenth of all deaths worldwide, especially in South Asia and Sub-Saharan Africa [3]. Likewise, in the low- and the middle-income countries, diarrhoea contributes to a major burden of disease [4].

Nepal has a population of almost 26.5 million with more than 80 % of the population residing in the rural area. More than one third (38 %) of the people do not have access to toilet facilities and about 18 % population do not have access to safe drinking water [5]. The under-five mortality and infant mortality are 54 and 46 deaths per 1000 live births, respectively [6].

Diarrhoea affects a large proportion of the under-five children of Nepal with increasing prevalence and a high annual incidence [7]. As per the Nepal Demographic and Health Survey (2011), the prevalence diarrhoea in under-five children of Nepal is 14 % [6]. This percentage is an increase from 12 % as found in the previous survey in 2005. Mortality among the under-five children with diarrhoea is still high in Nepal [8]. The consequences of diarrhoeal diseases range from poor growth, malnutrition and increase susceptibility to other infectious diseases and even deaths. The occurrence of diarrhoea among the under-five children worldwide has been linked mainly with hygiene and sanitation [9]. From the published literatures found on PubMed and Google Scholar search, we did not come across a structured analysis of available in a single research paper. We have a community-based approach for teaching and learning activities for medical and public health students at the B.P. Koirala Institute of Health Sciences, Nepal. We see a need for a comprehensive analysis of existing literatures on the different eco-social and behavioural aspects of diarrhoea in under-five children of Nepal. This result of the study is expected to help the students explore the communities to address these factors during their field research work and help the institute deliver community-based services to improve the health of the children. For this analysis, a framework given by Dahlgren and Whitehead [10] was appropriate to guide a structured analysis of the identified factors. We conducted this framework analysis to identify the eco-social and behavioural determinants of diarrhoea among the under-five children of Nepal.

## Materials and methods

**Fig. 1:**
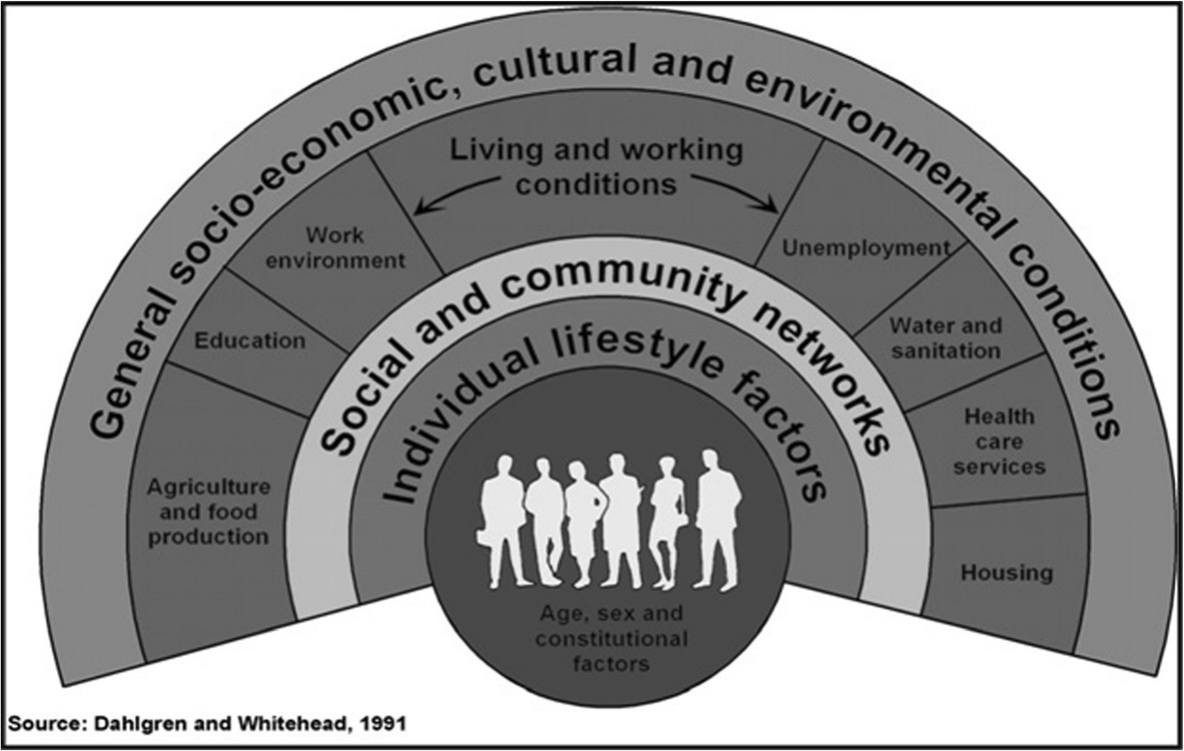
Dahlgren and Whitehead’s social model of health. This model explains the social theory of health mapping the relationship between the individual and the environment leading to health and disease

A rapid literature review was done for exploring the eco-social and behavioural determinants of diarrhoea in under-five children of Nepal. The review was done from June 2015 to October 2015. In literature searches, we did not find a review in the context of Nepal. With limited time and resources at hand, the rapid review method was chosen as we wanted to summarise the findings of Nepal in order to contribute to the teaching and learning activities for medical and public health students at the community level regarding childhood diarrhoea. The reference model chosen for this review is the Dahlgren and Whitehead model [10] (Fig. 1). The model was chosen, as it has stratified various determinants, which gives space for the exploration of diarrhoea as a disease of public health importance.

This paper explores the determinants of diarrhoea from an eco-social and behavioural perspective using the Dahlgren and Whitehead (1991) model of determinants of health. The model explains the different layers of influence on the health of an individual [10]. This model describes a social ecological theory to health and aims to map the relationship between the individual, their environment and the disease. Individual characteristics such as age and sex are at the centre of the model. The second layer includes the personal behaviour and ways of living that can promote or damage the health. The third layer is the social and community influences, i.e. social interaction in the local community. The fourth layer includes the structural factors: housing, working conditions, access to services and provision of essential facilities. The outermost layer talks about the general socioeconomic, cultural and environmental conditions.

The categories and keywords were used based on this model. The keywords used were ‘Diarrhoea’, ‘Determinant’, ‘factor’, ‘cause’, ‘under five’, ‘children’ and ‘Nepal’. With different combinations, the literatures were searched in PubMed, Nepal Journals Online and Google Scholar. As this is a rapid review, we also had to limit our search database yet to produce a comprehensive analysis of the factors. Among the popular databases for health sciences, PubMed and Google Scholar were chosen as they are also free search databases. The Nepal Journals online was used, as we are looking for publications relating to Nepal. Other databases like EMBASE and CINAHL could not be used as they require paid subscription which we do not have. Annual reports and reports of the Government of Nepal on health were also searched.

The oldest peer-reviewed articles found in our search was from 1989, so the inclusion criteria was set as all published articles reporting on causes, factors and practices of diarrhoea in under-five children in Nepal published between 1989 and July 2015. We set the lower limit for the year of publication of the articles in this as 1989, as we wanted to include all published literature with the above criteria from Nepal. All articles that reported on the clinical, microbial causes of diarrhoea were excluded, as our intention was to identify the eco-social and behavioural determinants of diarrhoea based on the Dahlgren and Whitehead model.

**Fig. 2:**
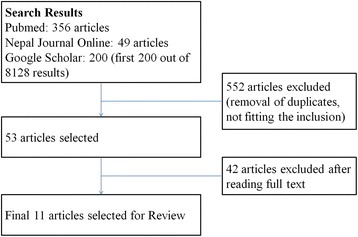
Articles selection process

After selection of the articles using the criteria set above, 11 peer-reviewed articles was used for the review. As a reference of the national data, we used the Nepal Demographic and Health Survey 2011 and Nepal Population and Housing Census 2011 published by the Government of Nepal. The article selection process is shown in Fig. 2.

During the discussion, some factors like unemployment, work environment, housing, agriculture and food production, social and community networks and hereditary factors are not mentioned as literatures on these topics were not found in relation to diarrhoea in Nepal.

In the paper, the results and discussion are under the same section; for each determinant, the first paragraph reports the results and the second paragraph has some discussion about the same determinant. The paper ends with a conclusion and a list of references.

## Results and discussion

The eco-social and behavioural determinants of diarrhoea among under-five children in Nepal are identified as follows: three factors related to the under-five children, two factors related to the mother and four broad social and environmental factors in this review.

The eco-social and behavioural determinants are listed in Table 1.

**Table 1:** Eco-social and behavioural determinants

Factors related to the under-five children	
• Age of the child [6, 11–13] • Gender of the child [6, 16, 33] • Nutritional status of children [6, 13]	
Factors related to mother	
• Hand-washing practice [19, 20] • Education [6]	
Social and environmental factors	
• Water and sanitation [11] • Healthcare services [6, 29] • Cultural and societal value [6, 13, 24, 32–34] • Household income [5, 6, 11]	

The details of the articles included in this review with key findings are presented in Table 2.

**Table 2:** Summary table of articles in this review

Sn	Author, year	Study details	Key findings
1	Joshi A, Shrestha RPB, 2012 [11]	Cross-sectional study involving 100 children of under 5 years	• Children between 6 and 23 months of age are more susceptible to diarrhoea. • Bloody diarrhoea was more common in children between 12 and 23 months of age. • Unsafe drinking water and lack of sanitary toilet are associated with childhood diarrhoea. • Occurrence of diarrhoea was lesser among children from higher wealth quintiles.
2	Ansari S, Sherchand JB, Parajuli K, Paudyal BM, Adhikari RP, Shrestha S et al., 2012 [12]	Cross-sectional study involving 525 children of under 5 years	• Occurrence of diarrhoea was more in children under 24 months. • Parasitic infection was higher among children between 6 and 24 months.
3	Strand TA, Sharma PR, Gjessing HK, Ulak M, Chandyo RK, Adhikari RK et al., 2012 [13]	Randomised controlled trial involving 335 children of 6–35 months of age	• Children between 6 and 23 months are more at risk for diarrhoea. • Children that are not breastfed are more at risk for diarrhoea occurrence and the extended duration of the episode.
4	Pokhrel S, Snow R, Dong H, Hidayat B, Flessa S, Sauerborn R, 2005 [16]	Secondary data review from national survey involving 8112 children under 15 years of age	• Male children are more likely to be taken healthcare during illness.
5	Langford RM. 2009 [19]	Community trial (PhD thesis); research included 88 children between 3 and 12 months	• Hand-washing practice among mothers was low after using the toilet, cleaning the child’s bottom, before handling food and before feeding the child. • Mothers washed hands only when visible contamination of faecal matter was seen. • Some mothers preferred to just wipe off the dirt without washing hands.
6	Rhee V, Mullany LC, Khatry SK, Katz J, LeClerq SC, Darmstadt GL, et al. 2008 [20]	Cohort study involving 23,662 newborns	• Mortality risk was lower among newborn whose mother or attendant washed hands regularly.
7	Ansari M, Ibrahim MIM, Shankar PR, 2011 [24]	Cross-sectional study involving 130 mothers with children of <5 years	• Mother with higher education had better knowledge on prevention of diarrhoea. • Mothers unaware about the association of the use of toilet and drinking safe water with the occurrence of diarrhoea.
8	Curtale F, Siwakoti B, Lagrosa C, LaRaja M, Guerra R, 1995 [29]	Comparative cross-sectional study involving 1443 mothers with children of <5 years of age and 208 community health volunteers	• Trained community health volunteers were effective in control of diarrhoea among the under-five children.
9	Ansari M, Izham M, Ibrahim M, Hassali MA, Shankar PR, 2011 [32]	Qualitative study involving 20 mothers	• Supernatural causes (witchcraft) linked with occurrence of diarrhoea among children. • Some mothers believe in the use of traditional healers for treating diarrhoea. • Lack of cleanliness was linked with diarrhoea by the mothers.
10	Ansari M, Palaian S, Ibrahim MIM, 2009 [33]	Review	• Cultural belief of supernatural powers in childhood diarrhoea exists in Nepal. • Traditional practice of fluid restriction and decreased breastfeeding during diarrhoea and use of herbs added to prolong the diarrhoeal episodes among children in Nepal.
11	Stapleton MC, 1989 [34]	Cross-sectional study among 320 health and developmental workers	• Beliefs regarding types of food causing diarrhoea prevailed in the community. • The concept of ‘hot’ food and ‘cold’ food linked with diarrhoea. • Evil spirits, frights, bad spells or gods were also linked with the occurrence of diarrhoea.

### Factors related to the under-five children

#### Age of the child

The under-five children of age group 6 to 23 months are more susceptible to diarrhoea [11, 12]. The prevalence of diarrhoea in this age group was found to be 48 %. A subgroup of age 12 to 23 months also had a higher prevalence of bloody diarrhoea compared to other age group [6, 11]. Children of age group 6 to 11 months and 12 to 23 months are at higher risk for illness duration due to diarrhoea in Nepal, compared to older children [13].

Supplementary diets are introduced to an infant around the age of 6 months in Nepal [14, 15]. Some mothers start weaning even before the age of 6 months [15].

#### Gender of the child

Gender has a role in healthcare seeking action from the reporting of the illness up to making a choice of the healthcare provider and the amount of money to be spent for seeking good health for children in Nepal [16]. Male children were more likely than female to be taken to a health facility for treatment [6].

There are disparities in decision-making for a male child and female child. Seeking healthcare is taken as an investment, so the parents prefer investing in male child as the society predominantly prefers son to a girl child especially the poor households [16, 17].

#### Nutritional status of children

In Nepal, 41 % of children under 5 years are stunted, 11 % are wasted and 29 % are underweight [6]. The chance of occurrence of diarrhoea is high in children with malnutrition [13]. The current high prevalence of malnutrition in Nepal may have played a significant role in the increase of diarrhoeal disease. Improving the nutritional status of children may reduce the incidence of diarrhoea and its persisting nature [13].

Malnourished children also have a higher chance of developing persistent diarrhoea, and once the diarrhoea begins, underweight children have a higher risk of their diarrhoeal episode becoming persistent [18].

### Factors related to mother

#### Hand washing by mothers

The practice of hand washing after touching faeces was not regular among the mothers in Nepal. Some mothers wash hands only when there is visible contamination with faecal matter. Some mothers just rub it off or just wipe it off [19]. Hand washing with soap and water is seen to be protective of diarrhoeal diseases, thereby preventing neonatal mortality in children [20]. Hand-washing practice is still not universal in Nepal. A large proportion of mothers do not wash hands. When there are no visible contaminations, the mothers do not wash hands at all. Some reported to wash hands only with water without the use of soap [19].

A study in Eastern Nepal showed that only about 65 % of the community people practice proper hand washing [21]. The practice of hand washing with soap and water has a preventive effect on the contracting a diarrhoeal disease. Proper hand-washing practices can reduce the diarrhoeal episodes by more than one third [22, 23].

#### Education

The children of the mothers that are educated up to secondary level or higher were suffering less from diarrhoea in comparison to children from mothers with lesser education. Education of mother may have a role in diarrhoea occurrence among under-five children in Nepal [6]. Mother with higher levels of education has better knowledge on prevention of diarrhoea [24].

In general, the higher the education level of a mother, she is more knowledgeable regarding the health status of her children. The female literacy rate in Nepal is 57.4 % [6].

### Social and environmental factors

#### Water and sanitation

Inadequate coverage of safe water supply and sanitation coverage has a major contribution to diarrhoea among children in Nepal. The unhygienic environmental situation increases the risk of water and food contamination [11]. Lack of sanitary toilets and lack of protected water sources are major factors associated with diarrhoea in Nepal [11].

Provision of sufficient quantities of safe water to the people has been taken as one of the important factors in the prevention of diarrhoea [25]. More than one third of the people (38 %) do not have access to toilet in Nepal. There is about 18 % population not having access to safe drinking water, with only less than half of the population (47 %) having access to tap/piped water. Safe water and sanitation provision still need to be distributed to a significant proportion of the people [5]. Limited water availability and poor water quality for drinking and sanitation is a major reason for diarrhoeal disease outbreak [11, 26]. The risk of acquiring diarrhoeal disease was four times higher than the reference scenario of tube well with toilet for scenario tube well without toilet [27].

Access to clean water and sanitation is accepted as necessary for all people and is taken as a basic human right by the United Nations (UN). This highlights the importance of clean water and sanitation for the health and wellbeing and also for prevention of diarrhoeal diseases [28]. Occurrence of diarrhoeal diseases is associated very closely with the sanitation conditions and drinking water. More than half of diarrhoeal disease deaths are attributed to unsafe drinking water, inadequate sanitation and poor hygiene [9].

#### Healthcare services

The proportion of the children with diarrhoea that are taken to the health facility was 38 % in 2011 [6]. Training community health volunteers (CHVs) for the treatment of diarrhoeal disease were effective in control of diarrhoea [29].

In Nepal, only around 50 % of the delivery occur in the healthcare institution of the total expected pregnancies [8]. Community-based integrated management of childhood illness (CB-IMCI) has increased the access of the children with diarrhoea to the healthcare. Trained health volunteers and health workers can identify the danger signs early so that morbidities and mortalities can be prevented due to diarrhoea [30]. The total number of diarrhoea cases reported was 1,766,903 cases in the country for a year in 2012–2013. Out of which, 77.22 % of diarrhoeal cases who sought care in the public sector were treated at community level by community-based health workers [8]. The use of community health volunteers has been shown to be effective for the diarrhoea occurrence and control. The CB-IMCI programme has imparted positive impact on the skills and knowledge of the health workers, enabling them for better identification, classification and treatment of diarrhoeal diseases [30]. Training of community workers to improve maternal and child health has been seen to be effective in Nepal [31].

#### Cultural and societal value

Supernatural causes of diarrhoea and the use of traditional healing methods like the ingestion of local banana for the management of diarrhoea are among the cultural beliefs and practices of the mothers in Nepal [24, 32, 33]. Practice of mothers withholding fluids to the children during diarrhoea is reported from the community [6]. Breastfeeding practices have been linked with the occurrence of diarrhoea. Lack of breastfeeding among children is associated with the occurrence of diarrhoea in children [13]. Mothers seem to have their own beliefs regarding the nature of diarrhoea, severity, kind of home management needed and kind of food to be taken as well as to avoid during such episodes as well as methods of prevention of diarrhoea [34].

Mothers in Nepal have beliefs that diarrhoea is a part of the childhood experience caused by evil spirits [19, 33]. This perception has an inverse relation in washing hands as a preventive practice for diarrhoea. According to a study, 22.5 % of the respondents found to have practised to give the bark of *Sorea robusta* and curd during the period of illness with a belief that it can control diarrhoea as well [35]. People from the Tharu community of middle Terai in Nepal are bounded on traditional beliefs, prejudices, social values, culture and traditional practices influencing to care seeking practices during diarrhoea [35].

#### Income

Children from families within the highest wealth quintile were seen to be less likely to be suffering from diarrhoea in comparison to children from other wealth quintiles [6, 11]. A recent report states that 25 % of the population in Nepal still live below the poverty line [5].

In Nepal, the expected cost for the utilisation of healthcare services is determined by the income status of the household. The poorer household is seen to postpone the healthcare seeking based on the estimated cost. This perception of the severity influences the treatment-seeking behaviour, which further determines the severity and consequences of the illness on the health of the child [17].

### Interaction between the factors

As the current model emphasises that there are interactions between the layers affect the individual in the centre of the model [10], the vulnerability of the children in the age group of 6–23 months is further aggravated by the hand-washing practices and cultural practice and beliefs of the mother [22, 23, 36]. Introduction of weaning foods to infants puts them at risk as this will be affected food practice and hand hygiene as well. The cultural beliefs and hand-washing practices are influenced by the education level of the mothers. Gender and income both influence the treatment-seeking behaviour. Gender of the child was seen to determine healthcare seeking for children. Opting for healthcare action was taken as investment which was seen to vary among children depending on whether the child is male or female [17]. Hand-washing practices with soap which are protective are influenced by the cultural beliefs [35]. Provision of safe water and sanitation has direct link with the prevention and control of diarrhoea. Involvement of community health volunteers increases the access to the health system, thereby reducing the diarrhoeal burden in the community [30].

## Conclusions

The use of Dahlgren G and Whitehead M (1991) model in this literature review has identified important eco-social and behavioural determinants of diarrhoea in under-five children of Nepal. Factors related to the under-five children (Age, gender and nutritional status), factors related to mother (hand-washing behaviour and education of mothers) and eco-social factors (water and sanitation, healthcare services, cultural and societal values and income of the household) are the identified determinants for diarrhoea in under-five children of Nepal. Research to address these factors may be needed to design local interventions to improve the morbidity and mortality due to diarrhoea in under-five children of Nepal.

### Limitations

The paper has limitations on literatures found, as literatures on researches done in Nepal were found in very limited number using the search engines and the key words as mentioned in the methodology. Some factors like unemployment, work environment, housing, agriculture and food production, social and community networks and hereditary factors are not discussed as literatures on these topics were not found in relation to diarrhoea.
